# Erie County Medical Veterinary Society

**Published:** 1894-09

**Authors:** C. J. Willgansz

**Affiliations:** (per J.) Secretary


### ERIE COUNTY VETERINARY MEDICAL SOCIETY.

At a meeting held at the office of Dr. N. P. Hinkley in Buffalo, N. Y., Wednesday evening, March 28, 1894. for the purpose of organizing a veterinary medical society for the County of Erie, the following practitioners of veterinary medicine were present:

Drs. J. Wende, Hinkley, Huessler, Ayers, Robinson, S. Somerville, Jr., B. Wende, Thompson, Block, Willyoung, McLeod, Gangloff, Willgansz and Wieland.

The meeting was called to order by Dr L. E. Willyoung, who said:

"*Gentlemen*:—At the meeting of the New York State Veterinary Medical Society, held in Syracuse in January, 1894, the subject of organizing county veterinary societies for the different counties throughout the State was discussed at length. While New York and Brooklyn have each a county society, there are no others at present in the State. As officers of the State society, we took it upon ourselves to organize and establish a permanent Veterinary Medical Society for the County of Erie, with objects as follows:

"*First.* To hold regular meetings for the purpose of discussing professional subjects, for mutual improvement, and to improve the status of the veterinary profession by bringing its members into more intimate relationship and consolidating them as a distinctive professional body in the community.

"*Second.* Members to consist of (*a*) Charter Members—all those practicing veterinary medicine who assist in the organization of the above association and conform to the regulations formulated by a two-thirds vote of those in attendance at the first meeting of the organization; (*b*) Future Members—to be elected, who shall be graduates of veterinary colleges which are legally entitled to grant veterinary diplomas, and which shall be recognized by this association as properly qualified, equipped and conducted for the same.

"Applications for future membership shall furnish: 1. Proof of graduation from one of such qualified institutions. 2. Vouchers signed by two members of the association, testifying to their reputable standing as business men and professional methods. 3. Shall be elected as provided for by the by-laws of this association.

"Gentlemen, we have issued invitations to every veterinary surgeon registered at the Erie County Clerk's office, and would be pleased to have every one become a member of the organization. The practitioners of human medicine have a county medical society which has been in existence for several years, and they have been fortunate enough to secure legislation which in a measure enables them to control the practice of medicine in this county. It protects its members, encourages and benefits all new-comers, and prevents impostors. In fact, in order to practice in this county, a physician must first join the society. With the progress we are making, the attention and respect we meet with from our Legislature, how long will it be before our society shall be vested with the same power?

"Now, gentlemen, I would suggest that we appoint or elect a chairman for this meeting."

Dr. John Wende was called to the chair, and Dr. Willyoung acted as Secretary.

It was decided by a two-thirds vote of those present that the name of the new association should be the Erie County Veterinary Medical Society.

It was decided to hold the privilege of joining open to non-graduates till the next meeting. The election of officers was then taken up and resulted as follows:

*President*—John Wende, V.S.

*Vice-President*—L. Robinson, V. S.

*Secretary*—C. J. Willgansz, V.M.D.
*Treasurer*—E. J. McLeod, D.V.S.

On motion of Dr. Willyoung the following committee on by-laws was appointed with instruction to report at the next meeting: Drs. Willyoung, S. Somerville, Jr., and Block, with the President and Secretary.

The meeting adjourned to meet again April 11th, at 8 P. M., at Dr. Hinkley's office.

C. J. WILLGANSZ, V.M.D.,
(per J.) Secretary.

